# Supplementary material for: Tissue-Specific Human Extracellular Matrix Scaffolds Promote Pancreatic Tumour Progression and Chemotherapy Resistance
Source: Cells. 2022 Nov 17;11(22):3652. doi: 10.3390/cells11223652 (PMC9688243; doi:10.3390/cells11223652)
Supplement: Supplementary file 1 [file cells-11-03652-s001.zip › cells-1957624-supplementary.pdf]

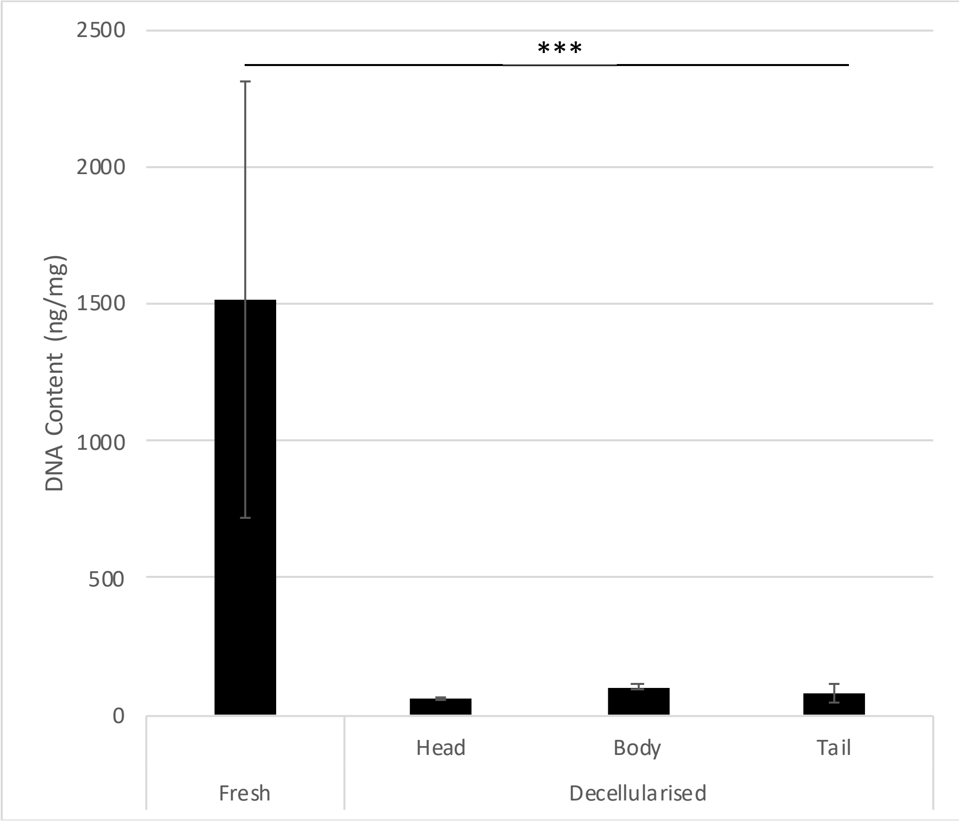

Supplementary Figure S1. DNA quantification of decellularised pancreas. DNA quantification showing significant elimination of DNA in the decellularised pancreas in all three segments: head, body and tail. Data are expressed as mean ± s.d. ***p < 0.0001

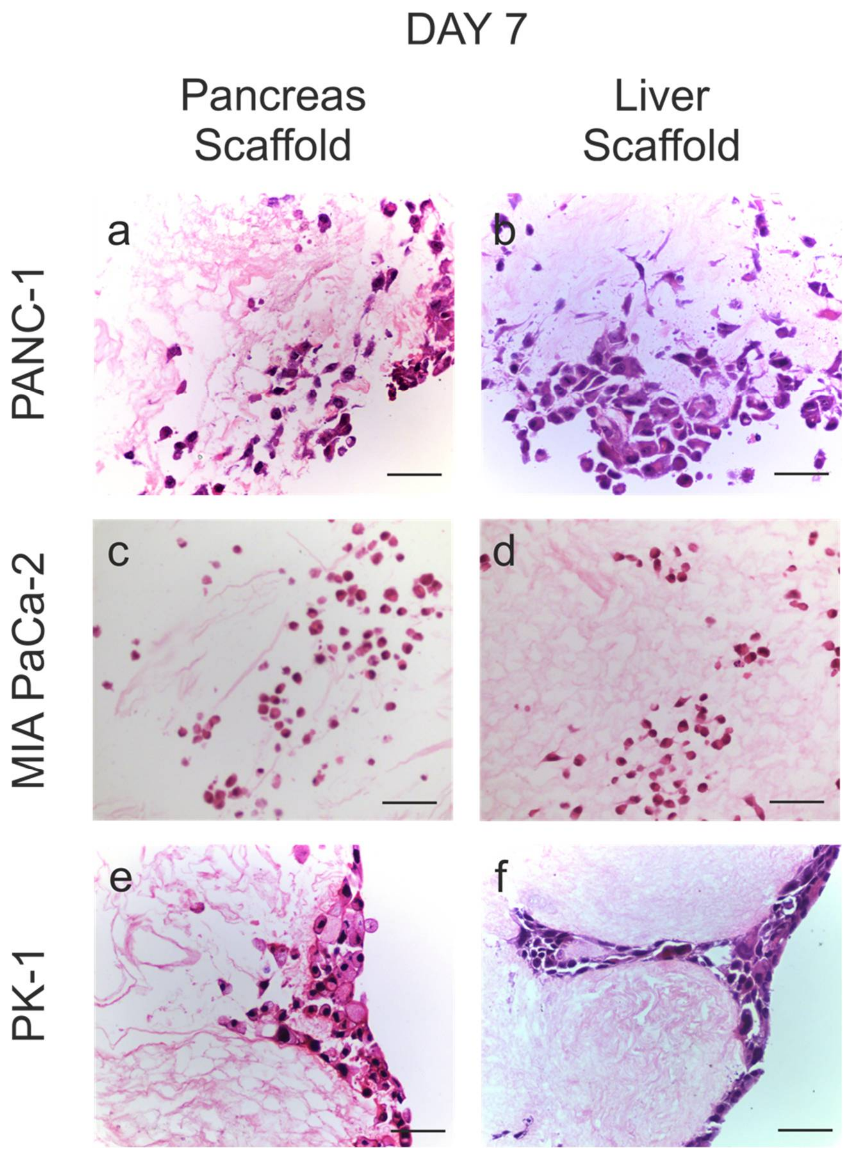

Supplementary Figure S2. PDAC cells on 3D scaffolds at Day 7 of culture. H&E staining of PDAC cells cultured for 7 days on decellularised scaffolds showed (a) PANC-1 cells on pancreas scaffolds and (b) PANC-1 cells on liver scaffolds both attached on the edge of the ECM. (c) MIA PaCa-2 cells on pancreas scaffolds and (d) MIA PaCa-2 cells on liver scaffolds both migrated into the ECM. (e) PK-1 cells on pancreas scaffolds only attached to the edge of the ECM and (f) PK-1 cells on liver scaffolds invaded the vessels and migrated into the ECM as thick clusters. Scale bars are representative of 50 μm.

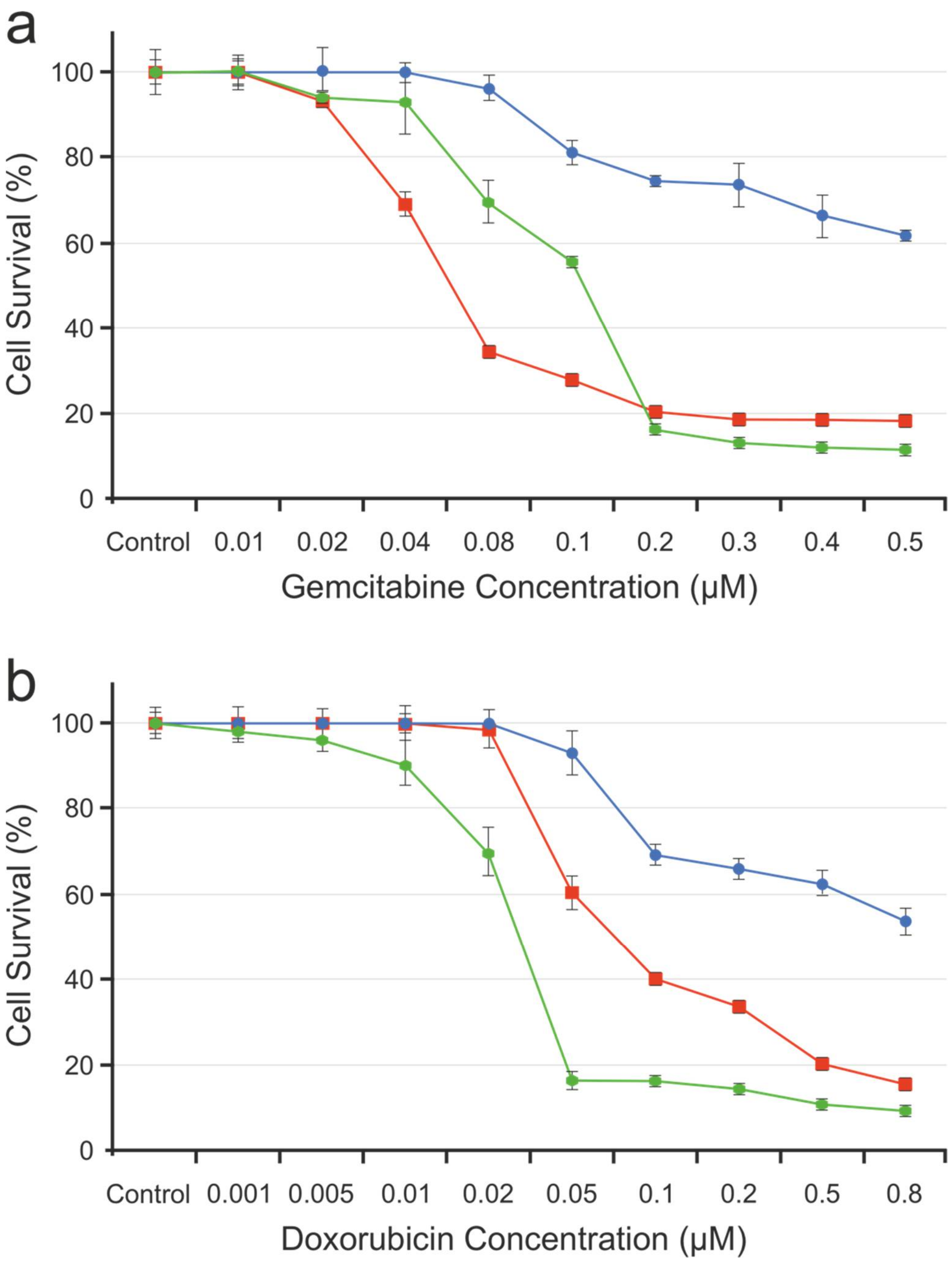

Supplementary Figure S3. 2D cultured PDAC cells treated with chemotherapeutics. PANC-1 (blue), MIA PaCa-2 (green) and PK-1 (red) cells were cultured on 2D plastic and treated with a series of incremental concentrations of (a) Gemcitabine and (b) Doxorubicin.

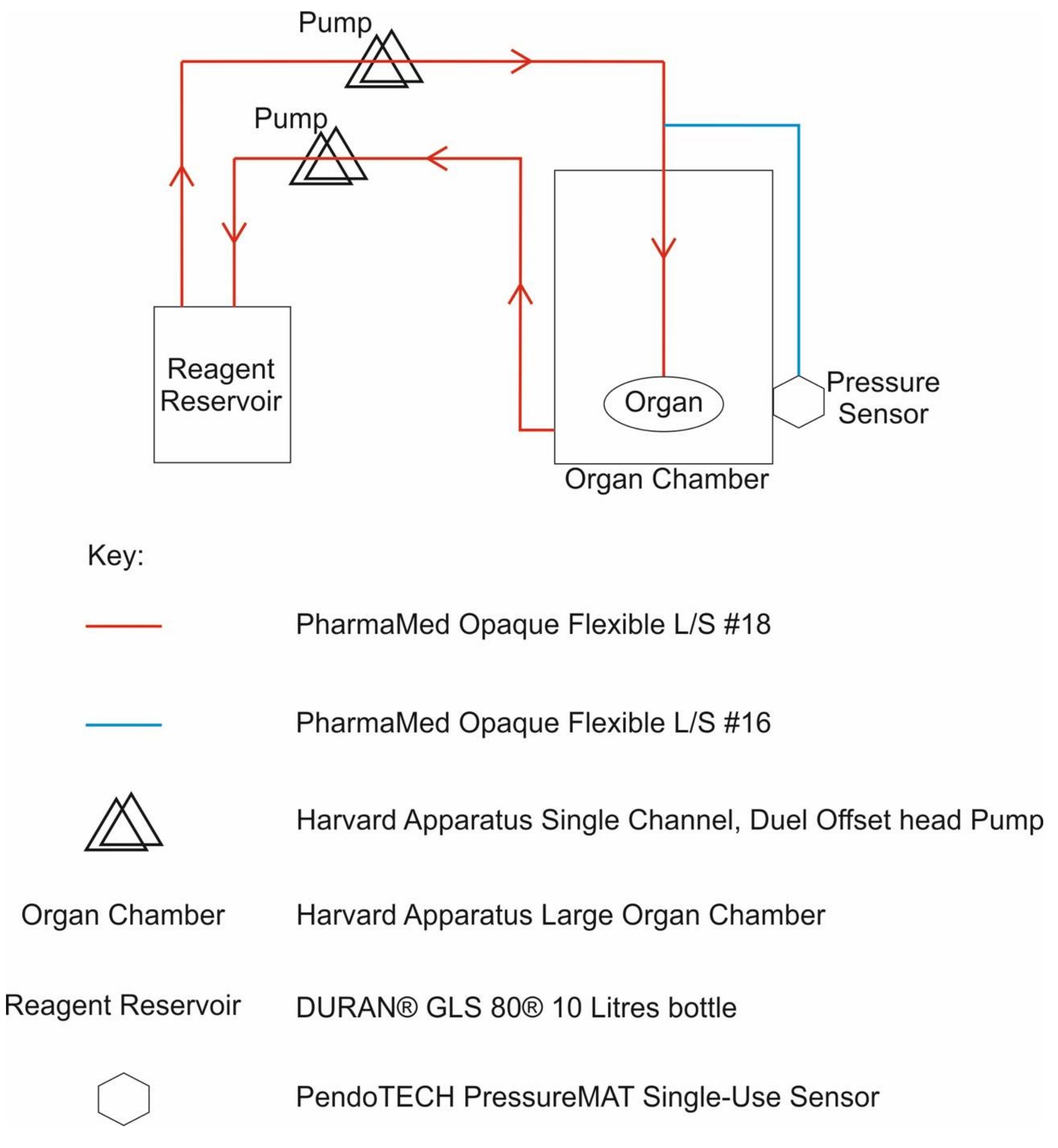

Supplementary Figure S4. Bioreactor set up for the decellularisation of whole human pancreata by perfusion.

**Step**	**Reagents**	**Time**	**Repetition**
1	Deionised Water (Milli-Q)	2	Repeat until solution is clear
2	Reagent Mixture	2	Repeat until tissue is clear
4	1% PBS solution	5	3

Supplementary Table S1. Decellularisation protocol for human liver cubes by agitation.

Day	Reagent	Mode of perfusion	Time (hours)	Pressure (mmHg)
Day 1	Distilled Water (Milli-Q)	Non-Recycle	2.5	5
	0.1 % SDS solution	Non-Recycle	0.5	5.5
	Distilled Water	Non-Recycle	1	6.5
	0.1 % SDS solution	Recycle	Overnight	7.5
Day 2	Distilled Water	Non-Recycle	1	10
	1% SDS solution	Recycle	1	10
	Distilled Water	Non-Recycle	1	10
	1% SDS solution	Recycle	Overnight	10
Day 3	1% SDS solution	Non-Recycle	1	15
Increase pressure to 20 mmHg and continue perfusion for 0.5 hours				
	Distilled Water	Recycle	2.5	20
	1% SDS solution	Recycle	Overnight	20
N.B. If the outflow at the start of day 4 is still cloudy, repeat all day 3 steps for another day				
Day 4	Distilled Water	Non-Recycle	1.5	30
	3% Triton X-100 solution	Recycle	Overnight	30
Day 5	Distilled Water	Non-Recycle	1.5	32.5
	3% Triton X-100 solution	Recycle	4	32.5
	Distilled Water	Non-Recycle	0.5	32.5
	3% Triton X-100 solution	Recycle	Overnight	32.5
Day 6	Distilled Water	Non-Recycle	1.5	35
	Distilled Water	Recycle	3.5	35
	3% Triton X-100 solution	Recycle	Overnight	35
N.B. If the outflow at the start of day 7 is still cloudy, repeat all day 6 steps for another day				
Day 7	Distilled Water	Non-Recycle	1	40

	Distilled Water	Recycle	4	40
	Distilled Water	Recycle	Overnight	40
Day 8	Distilled Water	Non-Recycle	1	40
	Distilled Water	Recycle	4	40
	Distilled Water	Recycle	Overnight	40
Day 9	1% PBS	Recycle	6	30
	1% PBS	Recycle	Overnight	30
Day 10	1% PBS	Recycle	6	30
	1% PBS	Recycle	Overnight	30
Day 11	PAA solution	Recycle	1	15
	1% PBS (Sterile)	Recycle	2	30

Supplementary Table S2. Decellularisation protocol for whole human pancreata by perfusion.

Supplementary Methods

Sample Processing for Histology and Immunohistochemistry

Fresh, decellularised and bioengineered tissue samples, previously fixed in 4% formaldehyde, were retrieved, dehydrated in a series of industrial IDA (Acquascience) and xylene (Acquascience) baths and finally embedded in paraffin. The samples were sliced into 4 µM sections using a Leica RM2035 microtome (Leica biosystems). All sections were then passed through three histology grade xylene baths for a minimum of 5 minutes, and then through three IDA baths for a minimum of 2 minutes, finally ending up in tap water (39).

DNA Quantification

Fresh and decellularised tissue samples marked for DNA quantification were retrieved from the -80 °C freezer and thawed in a 37 °C water bath for 1 h. The cubes were then weighed and if necessary, cut to be between 15 and 25 mg in mass. The cubes were then placed in 1.5 mL microcentrifuge tubes. Twenty µL of proteinase K was added to each, and then mixed thoroughly using a vortex. The cubes were then placed into a heating block at 56 °C for at least 16 h or until they were completely lysed. The DNA was then extracted using the QIAGEN DNAeasy Blood and Tissue Kit according to the manufacturer's instructions. The extracted DNA was eluted in 200 µL of buffer AE and was quantified using a NanoDrop ND-2000 spectrophotometer.

Drugs Preperation

Gemcitabine: A stock solution of 1 mM was prepared by solubilizing 0.026% (w/v) of gemcitabine powder (Gemzar) in distilled water. To obtain the desired drug concentrations, the stock solution was diluted in culture media.

Doxorubicin: A stock solution of 1 mM was prepared by solubilizing 0.058% (w/v) of doxorubicin hydrochloride powder (Sigma-Aldrich) in DMSO (Sigma-Aldrich). To obtain the desired drug concentrations, the stock solution was diluted in culture media.

Treatment of 3D Bioengineered Scaffolds with Chemotherapeutics

Both liver and pancreas scaffolds were prepared and seeded with PANC-1, MIA PaCa-2 or PK-1 cells as described above and cultured for 9 days. On the 9th day the media was discarded and 1.4 mL of 0.5 µM gemcitabine or 0.5 µM doxorubicin was added to the appropriate scaffolds. As a negative control, 1.4 mL of media was added to their respective scaffolds. The scaffolds were then allowed to incubate in the dark for 24 h in a humidified incubator at 37 °C with 5% CO_2. Next, the scaffolds were moved to fresh wells and washed with 1.4 mL 1X HBSS for 5 minutes and 1.4 mL of fresh media was added to each scaffold. The scaffolds were then left for 96 h in a humidified incubator at 37°C with 5% CO_2, to assess cell viability post-treatment using the Alamar Blue assay.

2D Cell Viability Assays

For experiments performed on 2D plastic, culture media was discarded, and the cells were washed three times with 200 µL 1X HBSS. Residual HBSS was discarded and 200 µL of a 10% Alamar Blue solution (Thermofisher Scientific) in culture media was added to each well. The cells were allowed to incubate with the Alamar Blue in the dark for 2.5 h in a humidified incubator at 37°C with 5% CO_2.

Fluorescence was measured immediately after incubation on a FLUOstar Omega fluorescence microplate reader (BMG Labtech) and quantified using excitation and emission wavelengths of 540 nm and 595 nm, respectively. The data measured in arbitrary units for the treated samples were normalized to the negative control (non-treated samples) and reduction in percent (%) survival was calculated.

Confirmation of Doxorubicin Uptake

To confirm the uptake of the chemotherapeutic agent by the cells in 3D scaffolds, doxorubicin's fluorescence was used. PANC-1 and MIA PaCa-2 cells on pancreas scaffolds and PK-1 cells on liver scaffolds were cultured for 9 days as described above. On day 9, the media was discarded and 1.4 mL of a 0.5 μM doxorubicin solution in cell culture media or fresh media was added to 3 samples of each condition followed by incubation in the dark for 24 h in a humidified incubator at 37 °C with 5% CO_2. Next, the scaffolds were washed with 1X PBS for 5 minutes, transferred to a mould with OCT (Agarscientific) and snap frozen in liquid nitrogen. Twenty micrometer sections were cut using a CRYOTOME FSE cryostat (Thermofisher Scientific). All sections were then washed 3 times for 5 minutes with 1X PBS and stained with 300 nM DAPI (Thermofisher Scientific) for 1 minute. Slides were imaged with a BX63 fluorescence microscope (Olympus) using excitation/emission of 350/460 nm (DAPI), 495/525 nm (collagen) and 545/620 nm (doxorubicin). Images were then processed with the software Fiji (ImageJ 1.52i).

RNA Extraction

Total RNA was extracted from 3D cultures using TRIzol reagent (Qiagen) and RNeasy Universal Mini Kit (Qiagen) as described by the manufacturer's instructions. Briefly, 3D frozen samples were left to incubate at room temperature with 650 μl of TRIzol in a 2 mL safe-lock Eppendorf tube for 20 minutes and was followed by the addition of 7 mm stainless steel

bead. The content was then agitated at 30 Hz for 8 minutes in a TissueLyser II (Qiagen). The content of the tube (excluding the bead) was then transferred to a new 1.5 mL Eppendorf tube and the manufacturer's protocol was followed from step 4.
